# Nontuberculous Mycobacterial Infections at a National Cancer Institute–Designated Comprehensive Cancer Center in Florida

**DOI:** 10.1093/ofid/ofag467

**Published:** 2026-07-29

**Authors:** Yanina Pasikhova, Matthew Snyder, Austin Morrison, Guy Handley, Misbahuddin Syed, Anthony P Cannella

**Affiliations:** Department of Pharmacy, Moffitt Cancer Center, Tampa, Florida, USA; Department of Pharmacy, Moffitt Cancer Center, Tampa, Florida, USA; Department of Pharmacy, Moffitt Cancer Center, Tampa, Florida, USA; Department of Infectious Diseases, Infection Control and Employee Health, The University of Texas MD Anderson Cancer Center, Houston, Texas, USA; Department of Internal Medicine, Moffitt Cancer Center, Tampa, Florida, USA; Division of Infectious Diseases and International Medicine, Department of Internal Medicine, University of South Florida Morsani College of Medicine, Tampa, Florida, USA

**Keywords:** dual β-lactam, nontuberculous mycobacteria, oncology

## Abstract

**Background:**

Nontuberculous mycobacteria (NTM) are environmental organisms that become opportunistic pathogens in certain populations. NTM prevalence has increased globally, likely due to increased diagnostic testing and the availability of population-based studies. Current literature on oncology patients is minimal and represents a unique study area. Here we present the epidemiology and treatment of NTM infection cases at a National Cancer Institute–designated comprehensive cancer center.

**Methods:**

We performed a retrospective single-center cohort study of adults with microbiologically proven NTM infections presenting from 2020 to 2023. Epidemiologic and treatment-related data were retrieved from the electronic medical record and evaluated to illustrate institutional patterns.

**Results:**

A total of 93 patients met assessment criteria, with most (74 of 93 [80%]) having a past or active cancer. *Mycobacterium avium* complex (47 of 93 [51%]) and *Mycobacterium abscessus* group (28 of 93 [30%]) were the most common isolates. The majority were pulmonary infections (75 of 93 [81%]), with the integument accounting for most extrapulmonary sites (14 of 18 [78%]). Of 93 patients, 49 (53%]) received therapy, with many (41 of 49 [84%]) receiving ≥3 agents. Thirty-nine (80%]) required an initial regimen modification, and 13 (27%) received dual β-lactam therapy (frequently including meropenem). Of treated patients, 36 (73%) demonstrated improved symptoms, 18 (37%) improved or partially improved radiologic change, and 19 (39%]) sustained culture conversion to negative at the evaluation time point; 13 (27%) died or were lost to follow-up.

**Conclusions:**

Our report offers one of the largest single-center epidemiologic perspectives on NTM infections in oncology patients. It offers unique insight into treatment practices in this population, particularly regarding dual β-lactam therapy.

The global incidence of nontuberculous mycobacteria (NTM) disease has significantly increased over the last 20 years [1]. Most cases are pulmonary in origin; however, extrapulmonary manifestations often present as integumental infections or disseminated disease in patients with immunodeficiencies or immunosuppression [1–6]. Data, globally and within the United States, have shown species within the *Mycobacterium avium* complex (*M avium*, *Mycobacterium chimaera*, *Mycobacterium intracellulare*) to be the most commonly isolated in NTM infections [1, 5]. However, the prevalence of species might vary geographically, as a marked increase in *Mycobacterium abscessus* group has been seen in East Asian countries and in the southeastern United States [1, 7]. More recently, a study conducted by members of our group at a tertiary central Florida medical center found *M abscessus* group (subspecies *abscessus*, *bolletii*, and *massiliense*) to have the highest prevalence [8]. This contrasts with a similar epidemiologic study from Oregon showing *M avium* complex as the most identified pathogen at the study facility [9]. This suggests that environmental conditions exist as a risk factor for prevalence of different NTM species. Pulmonary infections are most common, yet infections of skin and soft tissue, infections of prosthetic materials, and bacteremias also occur.

Over the past 20 years it has been apparent that more NTM cases are being diagnosed, with the thought being that there is an increase in diagnostic testing as well as availability of population-based studies. Currently there is a paucity of literature regarding NTM infections in oncology patients. This warrants a unique area for study, especially in a densely populated state such as Florida. Here our group presents an epidemiologic and clinical presentation of NTM infection cases at a National Cancer Institute–designated comprehensive cancer center in Florida.

## METHODS

This single-center cohort study was approved by the Institutional Review Board of the University of South Florida (STUDY004438) and the Moffitt Cancer Center Scientific Review Committee (MCC 21983). The Institutional Review Board determined that the study qualified for expedited review, in addition to a waiver of the requirements for the informed consent and signed authorization process. The study consisted of a retrospective review of patients presenting with NTM infections from May 2020 through April 2023 at the Moffitt Cancer Center, a National Cancer Institute–designated comprehensive cancer center in Tampa, Florida, associated with the University of South Florida Morsani College of Medicine. Data were abstracted from the health system's electronic medical record and recorded in a standardized electronic case report form.

Patients met the inclusion criteria if they were aged ≥18 years and were seen by the Moffitt Cancer Center Infectious Diseases/NTM clinic with NTM infection at any body site microbiologically confirmed by culture, polymerase chain reaction, or mass spectrometry within the designated time range (April 2023 or earlier). Exclusion criteria were defined as acid-fast bacilli microbiologic cultures collected from outside facilities (unless otherwise deemed sufficient by our providers) and belonging to a vulnerable population, including prisoners and pregnant women. Pulmonary microbiologic criteria were incorporated from a combination of specialist input and/or consensus guidelines, which included (1) positive cultures from ≥2 separate expectorated sputum samples, (2) positive culture results for NTM from ≥1 bronchial wash or lavage sample, and (3) transbronchial or other lung biopsy specimen with mycobacterial histologic features (granulomatous inflammation or acid-fast bacilli) and positive culture for NTM or biopsy showing mycobacterial histologic features (granulomatous inflammation or acid-fast bacilli) and ≥1 sputum or bronchial wash sample that are culture positive for NTM [10, 11].

Extrapulmonary criteria included positive culture results for NTM from samples of the affected site, as well as applicable clinical features and/or radiographic findings indicative of infection [12]. The clinical diagnosis of pulmonary or extrapulmonary disease was defined by expert-led guidelines, determined by an infectious diseases clinician with expertise in NTM, and based on clinical and radiologic presentations (pulmonary or systemic symptoms and nodular or cavitary opacities on chest radiographs or a high-resolution computed tomographic scan showing bronchiectasis with multiple small nodules) [10, 11]. True infection versus colonization (particularly for pulmonary cases) was delineated based on the characteristics of the organism identified in culture and most prominently in patient symptoms [10, 11]. Symptoms (which for pulmonary infections included chills, fever, night sweats, weight loss, cough, shortness of breath, and hemoptysis) not only had to be present but also had to fit the appropriate time course to be deemed attributable to NTM infection (along with other possible causes being excluded) [10, 11].

After confirmation of NTM infection based on physician assessment and consensus guidelines, the primary objective was to evaluate the clinical outcomes for patients receiving antimicrobial therapy [10–14]. Symptomatic change was determined at the discretion of the treating physician and labeled as either no change/unknown, improvement, persistent, or worsening. Radiologic change was determined by radiologist assessment of various imaging methods and categorized into unknown/too early to tell, improved, partially improved, unchanged, or worsened. Microbiologic change was assessed using applicable culture data and classified as either no change/unknown, sustained culture conversion to negative, culture conversion with late relapse, or continued culture positivity. Secondary analysis was performed on a number of prespecified safety outcomes, including the incidence of changes to the initial treatment regimen (and whether that occurred due to a treatment-related adverse event [a single drug change due to toxicity], tolerance issue [≥2 drug changes or therapy permanently held due to toxicity], or efficacy/optimization issue [change in any number of drugs to optimize treatment efficacy or due to logistical/assorted outside issues]) and occurrence of 30-day, 60-day, NTM-attributable, and all-cause deaths during the study time frame.

Demographic information collected included age, sex, race, height, weight, body mass index, and cancer history. Microbiologic characteristics of the active NTM infection during the study period were also recorded, including type of mycobacteria, site of infection and culture, and susceptibility data (minimum inhibitory concentration and susceptible/intermediate/resistant) for amikacin, macrolides, clofazimine, and imipenem-cilastatin. Aside from the objectives, other treatment-related details that were gathered included treatment status (surveillance/monitoring or treatment), agent(s) selected for initial treatment regimen, agent(s) switched to when the treatment regimen changed, and duration of therapy.

A descriptive statistical analysis was conducted to assess patients’ demographic characteristics. Median or mean values and ranges are provided for continuous variables, and patient numbers and percentages for nominal variables.

## RESULTS

Ninety-three cases of NTM infections met assessment criteria and were included in the study. Patient and microbiologic characteristics are shown in Table 1. Patients tended to be older (median age, 68 years), female (67 of 93 [72%]), and white (83 of 93 [89%]). Most patients had a past or active cancer (74 of 93 [80%]), with approximately a quarter (24 of 93 [26%]) having >1 type of cancer. Of the various cancer types, integument (21 of 93 [23%]), lung (20 of 93 [22%]), and breast (12 of 93 [13%]) cancers were most common. *M avium* complex (47 of 93 [51%]) and *M abscessus* group (28 of 93 [30%]) were the most frequent NTM isolates. The majority of infections were pulmonary (75 of 93 [81%]). The integument (14 of 18 [78%]) was the site of most extrapulmonary infections, followed by the bloodstream (3 of 18 [17%]) and sinus (1 of 18 [6%]). Table 2 shows site of infection/culture and type of cancer stratified by organism. All *M avium* complex cases (47 of 47 [100%]) were pulmonary, while *M abscessus* group infections were more evenly split between pulmonary (13 of 28 [46%]) and extrapulmonary (15 of 28 [54%]). *M avium* complex was the most common pathogen found in those with integument cancer (11 of 21 [52%]), lung cancer (14 of 20 [70%]), lymphoma (5 of 6 [83%]), head and neck cancer (3 of 5 [60%]), and bladder cancer (2 of 3 [67%]). *M abscessus* group was the most frequent isolate found in cases of leukemia (5 of 9 [56%]), breast cancer (7 of 12 [58%]), and multiple myeloma (3 of 5 [60%]), among others. Antimicrobial susceptibilities for the various isolates are shown in Table 3.

**Table 1.** Patient and Microbiologic Characteristics

Characteristic	Patients, No./Total (%)^a^
Age, median (range), y	68 (36–89)
Height, median (range), cm^b^	166 (149–188)
Weight, median (range), kg^b^	64 (38–123)
Boby mass index, median (range)^b,c^	23 (15–38)
Female sex	67/93 (72)
Race	
White	83/93 (89)
Latino	6/93 (6)
Black or African American	2/93 (2)
Other	2/93 (2)
No. of past or current cancers^d^	
0	19/93 (20)
1	50/93 (54)
2	18/93 (19)
3	6/93 (6)
Type of cancer^d^	
Integument	21/93 (23)
Lung	20/93 (22)
Breast	12/93 (13)
Leukemia	9/93 (10)
Lymphoma	6/93 (6)
Head and neck	5/93 (5)
Multiple myeloma	5/93 (5)
Prostate	4/93 (4)
Esophageal/esophagogastric junction	3/93 (3)
Sarcoma	3/93 (3)
Bladder	3/93 (3)
Pancreatic	3/93 (3)
Other^e^	11/93 (12)
*Mycobacterium* organism	
*M avium* complex	47/93 (51)
*M abscessus* group	28/93 (30)
*M kansasii*	2/93 (2)
*M chelonae*	1/93 (1)
*M fortuitum*	4/93 (4)
Other^f^	11/93 (12)
Site of Infection	
Pulmonary	75/93 (81)
Extrapulmonary	18/93 (19)
Bloodstream	3/18 (17)
Integument	14/18 (78)
Sinus	1/18 (6)
Site of culture	
Sputum	12/93 (13)
Smear	10/93 (11)
Bronchoalveolar lavage	57/93 (61)
Skin and soft-tissue infections/fluid	9/93 (10)
Bone	1/93 (1)
Blood	4/93 (4)

^a^Data represent no. of patients/total (%) except where otherwise specified.

^b^Height, weight, and body mass index data were not available for 1 patient.

^c^Body mass index was calculated as weight in kilograms divided by height in meters squared.

^d^Five patients had 2 different cases of the same type of cancer; these were counted as a single cancer.

^e^Other types of cancer included ovarian cancer, myelodysplastic syndrome, colorectal cancer, renal cell carcinoma, ampullary adenocarcinoma, myelofibrosis, hepatocellular cancer, penile cancer, and gastrointestinal stromal tumor.

^f^Other *Mycobacterium* isolates (≥1 per patient) included *M stomatepiae, M angelicum, M szulgai, M phocaicum, M mucogenicum, M intracellulare, M simiae, M asiaticum*, and polymicrobial isolates including *M fortuitum* with *M avium* complex in 1 patient; *M cosmeticum, M avium* complex, and *M abscessus* group in 1; and *M avium* complex and *M abscessus* group in 2.

**Table 2.** Site of Infection and Culture by Organism

Variable	Patients by *Mycobacterium* Species, No./Total (%)					
	*M avium* Complex (n = 47)	*M abscessus* Group (n = 28)	*M kansasii* (n = 2)	*M chelonae* (n = 1)	*M fortuitum* (n = 4)	Other^a^ (n = 11)
Infection site						
Pulmonary	47/47 (100)	13/28 (46)	2/2 (100)	1/1 (100)	2/4 (50)	10/11 (91)
Extrapulmonary	0/47 (0)	15/28 (54)	0/2 (0)	0/1 (0)	2/4 (50)	1/11 (9)
Bloodstream	NA	3/15 (20)	NA	NA	0/2 (0)	0/1 (0)
Integument	NA	11/15 (73)	NA	NA	2/2 (100)	1/1 (100)
Sinus	NA	1/15 (7)	NA	NA	0/2 (0)	0/1 (0)
Culture site						
Sputum	6/47 (13)	1/28 (4)	1/2 (50)	1/1 (100)	1/4 (25)	2/11 (18)
Smear	4/47 (9)	4/28 (14)	1/2 (50)	0/1 (0)	0/4 (0)	1/11 (9)
BAL	37/47 (79)	12/28 (43)	0/2 (0)	0/1 (0)	1/4 (25)	7/11 (64)
SSTI/fluid	0/47 (0)	6/28 (21)	0/2 (0)	0/1 (0)	2/4 (50)	1/11 (9)
Bone	0/47 (0)	1/28 (4)	0/2 (0)	0/1 (0)	0/4 (0)	0/11 (0)
Blood	0/47 (0)	4/28 (14)	0/2 (0)	0/1 (0)	0/4 (0)	0/11 (0)
Cancer type^b^						
Integument	11/21 (52)	7/21 (33)	0/21 (0)	0/21 (0)	1/21 (5)	2/21 (10)
Lung	14/20 (70)	2/20 (10)	0/20 (0)	1/20 (5)	1/20 (5)	2/20 (10)
Breast	4/12 (33)	7/12 (58)	0/12 (0)	0/12 (0)	1/12 (8)	0/12 (0)
Leukemia	3/9 (33)	5/9 (56)	0/9 (0)	0/9 (0)	0/9 (0)	1/9 (11)
Lymphoma	5/6 (83)	0/6 (0)	0/6 (0)	0/6 (0)	0/6 (0)	1/6 (17)
Head and neck	3/5 (60)	1/5 (20)	0/5 (0)	0/5 (0)	1/5 (20)	0/5 (0)
Multiple myeloma	2/5 (40)	3/5 (60)	0/5 (0)	0/5 (0)	0/5 (0)	0/5 (0)
Prostate	2/4 (50)	2/4 (50)	0/4 (0)	0/4 (0)	0/4 (0)	0/4 (0)
Esophageal/EGJ	1/3 (33)	2/3 (67)	0/3 (0)	0/3 (0)	0/3 (0)	0/3 (0)
Sarcoma	1/3 (33)	0/3 (0)	1/3 (33)	0/3 (0)	0/3 (0)	1/3 (33)
Bladder	2/3 (67)	1/3 (33)	0/3 (0)	0/3 (0)	0/3 (0)	0/3 (0)
Pancreas	1/3 (33)	1/3 (33)	0/3 (0)	0/3 (0)	0/3 (0)	1/3 (33)
Other^c^	6/11 (55)	3/11 (27)	0/11 (0)	0/11 (0)	0/11 (0)	2/11 (18)

Abbreviations: BAL, bronchoalveolar lavage; EGJ, esophagogastric junction; NA, not applicable; SSTI, skin and soft-tissue infection.

^a^Other *Mycobacterium* isolates (≥1 per patient) included *M stomatepiae, M angelicum, M szulgai, M phocaicum, M mucogenicum, M intracellulare, M simiae, M asiaticum*, and polymicrobial isolates including *M fortuitum* with *M avium* complex in 1 patient; *M cosmeticum, M avium* complex, and *M abscessus* group in 1 patient; and *M avium* complex and *M abscessus* group in 2 patients.

^b^Five patients each had 2 cases of the same type of cancer, which were counted as a single cancer.

^c^Other types of cancer included ovarian cancer, myelodysplastic syndrome, colorectal cancer, renal cell carcinoma, ampullary adenocarcinoma, myelofibrosis, hepatocellular cancer, penile cancer, and gastrointestinal stromal tumor.

**Table 3.** Susceptibility by Organism

*Mycobacterium* Organism	Drug	Isolates by MIC (in mg/L), No.															Isolates Tested by Susceptibility, No. (%)^a^		
																	Susceptible	Intermediate	Resistant
		0.03	0.06	0.12	0.25	0.5	1	2	4	5	8	9	16	32	64	128			
*M avium* complex (n = 47)	Amikacin	0	0	0	0	0	3	1	21	0	9	0	8	2	0	0	42 (95)	2 (5)	0 (0)
	Macrolide	0	0	5	18	5	7	6	0	1	0	0	0	1	0	1	42 (95)	0 (0)	2 (5)
	Clofazimine	2	6	13	9	0	0	0	0	0	0	0	0	0	0	0	NI	NI	NI
	Imipenem-cilastatin	0	0	0	0	0	0	0	0	0	1	0	0	0	0	0	0 (0)	1 (100)	0 (0)
*M abscessus* group (n = 28)	Amikacin	0	0	0	0	0	0	0	0	0	1	0	14	13	0	0	15 (54)	13 (46)	0 (0)
	Macrolide	0	0	1	2	3	10	2	2	0	2	1	4	1	0	0	18 (64)	2 (7)	8 (29)
	Clofazimine	0	0	0	1	22	0	0	0	0	0	0	0	0	0	0	NI	NI	NI
	Imipenem-cilastatin	0	0	0	0	0	0	0	0	0	14	0	7	3	2	0	0 (0)	21 (81)	5 (19)
*M kansasii* (n = 2)	Amikacin	0	0	0	0	0	0	0	0	0	0	0	0	0	0	0	NT	NT	NT
	Macrolide	0	1	0	0	1	0	0	0	0	0	0	0	0	0	0	2 (100)	0 (0)	0 (0)
	Clofazimine	0	0	0	0	0	0	0	0	0	0	0	0	0	0	0	NT	NT	NT
	Imipenem-cilastatin	0	0	0	0	0	0	0	0	0	0	0	0	0	0	0	NT	NT	NT
*M chelonae* (n = 1)	Amikacin	0	0	0	0	0	0	0	0	0	0	0	0	0	0	0	NT	NT	NT
	Macrolide	0	0	0	1	0	0	0	0	0	0	0	0	0	0	0	1 (100)	0 (0)	0 (0)
	Clofazimine	0	0	0	0	0	0	0	0	0	0	0	0	0	0	0	NT	NT	NT
	Imipenem-cilastatin	0	0	0	0	0	0	0	0	0	0	0	1	0	0	0	0 (0)	1 (100)	0 (0)
*M fortuitum* (n = 4)	Amikacin	0	0	0	0	0	2	2	0	0	0	0	0	0	0	0	4 (100)	0 (0)	0 (0)
	Macrolide	0	0	0	0	0	0	0	0	0	3	0	1	0	0	0	0 (0)	0 (0)	4 (100)
	Clofazimine	0	0	0	2	0	0	0	0	0	0	0	0	0	0	0	NI	NI	NI
	Imipenem-cilastatin	0	0	0	0	0	0	2	2	0	0	0	0	0	0	0	4 (100)	0 (0)	0 (0)
Other^b^ (n = 11)	Amikacin	0	0	0	0	0	2	2	0	0	2	0	1	0	0	0	7 (100)	0 (0)	0 (0)
	Macrolide	0	1	2	1	2	0	1	0	0	0	0	0	0	0	0	7 (100)	0 (0)	0 (0)
	Clofazimine	0	0	0	3	0	0	0	0	0	0	0	0	0	0	0	NI	NI	NI
	Imipenem-cilastatin	0	0	0	0	0	0	1	0	0	0	0	0	0	0	0	1 (100)	0 (0)	0 (0)

Abbreviations: MIC, minimum inhibitory concentration; NI, no interpretation; NT, none tested.

^a^Not all isolates were tested for susceptibility to each drug. Susceptibility percentages are reported as a proportion of the total number of isolates tested.

^b^Other *Mycobacterium* isolates (≥1 per patient) included *M stomatepiae, M angelicum, M szulgai, M phocaicum, M mucogenicum, M intracellulare, M simiae, M asiaticum,* and polymicrobial isolates including *M fortuitum* with *M avium* complex in 1 patient; *M cosmeticum, M avium complex,* and *M abscessus* group in 1 patient; and *M avium* complex and *M abscessus* group in 2 patients. Susceptibilities were not reported for polymicrobial cases.

Treatment-related characteristics can be viewed in Table 4. In total, 49 of 93 patients (53%) received treatment from our providers. Only 23 of 47 patients (49%) with *M avium* complex received therapy, compared with most patients with *M abscessus* group infections (19 of 28 [68%]). With regard to cancer type among those who received treatment, 26 of 49 patients (53%) had solid tumors, while only 18 of 49 (37%) had hematologic cancer. Of all 49 patients treated, 41 (84%) were started on regimens including ≥3 agents. Three-drug regimens were more commonly used as front-line therapy in patients with *M avium* complex (19 of 23 [83%]), *M abscessus* group (10 of 19 [53%]), pulmonary (25 of 34 [74%]), and extrapulmonary (6 of 15 [40%]) infections. The most prominent regimen used for *M avium* complex pulmonary infection by far was azithromycin, ethambutol, and rifabutin/rifampin (in 15 of 23 patients [65%]). Clofazimine was used upfront in 6 of 49 treated patients (12%) and most commonly in the management of patients with *M abscessus* group infections (5 of 6 [83%]).

**Table 4.** Treatment Characteristics

Variable	Patients, No./Total (%)^a^
Treatment approach	
Overall population	
Surveillance	44/93 (47)
Treatment	49/93 (53)
By organism	
*Mycobacterium avium* complex	
Surveillance	24/47 (51)
Treatment	23/47 (49)
*Mycobacterium abscessus* group	
Surveillance	9/28 (32)
Treatment	19/28 (68)
*Mycobacterium kansasii*	
Surveillance	1/2 (50)
Treatment	1/2 (50)
*Mycobacterium chelonae*	
Surveillance	1/1 (100)
Treatment	0/1 (0)
*Mycobacterium fortuitum*	
Surveillance	1/4 (25)
Treatment	3/4 (75)
Other^b^	
Surveillance	8/11 (73)
Treatment	3/11 (27)
By infection site	
Pulmonary	
Surveillance	41/75 (55)
Treatment	34/75 (45)
Extrapulmonary	
Surveillance	3/18 (17)
Treatment	15/18 (83)
No. of agents in initial regimen	
By organism	
*M avium* complex	
4 drugs	2/23 (9)
3 drugs	19/23 (83)
2 drugs	2/23 (9)
*M abscessus* group	
5 drugs	1/19 (5)
4 drugs	6/19 (32)
3 drugs	10/19 (53)
2 drugs	2/19 (11)
*M kansasii*	
3 drugs	1/1 (100)
*M chelonae*	NA^c^
*M fortuitum*	
2 drugs	3/3 (100)
Other^b^	
4 drugs	1/3 (33)
3 drugs	1/3 (33)
1 drug	1/3 (33)
Infection site	
Pulmonary	
4 drugs	4/34 (12)
3 drugs	25/34 (74)
2 drugs	4/34 (12)
1 drug	1/34 (3)
Extrapulmonary	
5 drugs	1/15 (7)
4 drugs	5/15 (33)
3 drugs	6/15 (40)
2 drugs	3/15 (20)
Most common initial regimen	
By organism	
*M avium* complex	
Azithromycin, ethambutol, and rifabutin/rifampin	15/23 (65)
*M abscessus* group	
Azithromycin, ceftaroline, meropenem, and tigecycline	2/19 (11)
*M kansasii*	
Azithromycin, ethambutol, and rifabutin	1/1 (100)
*M chelonae*	NA^c^
*M fortuitum*	
Ciprofloxacin and sulfamethoxazole-trimethoprim	1/3 (33)
Doxycycline and linezolid	1/3 (33)
Meropenem and moxifloxacin	1/3 (33)
Other^c^	
Azithromycin	1/3 (33)
Azithromycin, ethambutol, and rifabutin	1/3 (33)
Azithromycin, ceftaroline, imipenem-cilastatin, and tigecycline	1/3 (33)
By infection site	
Pulmonary	
Azithromycin, ethambutol, and rifabutin/rifampin	18/34 (53)
Extrapulmonary	
Azithromycin, ceftaroline, meropenem, and tigecycline	2/15 (13)
Notable use for initial regimen	
Carbapenem as part of dual β-lactam therapy	9/49 (18)
Imipenem-cilastatin (with ceftaroline or ceftazidime)	5/9 (56)
Meropenem (with ceftaroline)	4/9 (44)
Bedaquiline as part of initial therapy	1/49 (2)
* M abscessus* group involved with bedaquiline use	1/1 (100)
Clofazimine as part of initial therapy	6/49 (12)
Site of disease with clofazimine use	
Pulmonary	2/6 (33)
Extrapulmonary	4/6 (67)
Organism involved with clofazimine use	
*M avium* complex	1/6 (17)
*M abscessus* group	5/6 (83)
Rationale for modifications to treatment course	
Any modification^d^	39/49 (80)
Treatment-related adverse event (1 drug change)	8/49 (16)
Tolerance issue (≥2 drug changes or therapy permanently held)	22/49 (45)
Efficacy/optimization issue	16/49 (33)
Notable use at any point during entire regimen	
Carbapenem as part of dual β-lactam therapy^e^	13/49 (27)
Imipenem-cilastatin (with ceftaroline or ceftazidime)	7/13 (54)
Meropenem (with ceftaroline)	8/13 (62)
Clofazimine	9/49 (18)
Bedaquiline	2/49 (4)
Duration of therapy, median (range), d	
Total (all lines of treatment via any route for 49 patients receiving therapy)	147 (14–851)
Intravenous (intravenous therapy for 24 patients receiving agents via that route)	90 (25–655)

Abbreviation: NA, not applicable.

^a^Data represent no. of patients/total (%) unless otherwise specified.

^b^Other *Mycobacterium* isolates (≥1 per patient) included *M stomatepiae*, *M angelicum*, *M szulgai*, *M phocaicum*, *M mucogenicum*, *M intracellulare*, *M simiae*, *M asiaticum*, and polymicrobial isolates, including *M fortuitum* with *M avium* complex in 1 patient; *M cosmeticum*, *M avium* complex, and *M abscessus* group in 1 patient; and *M avium* complex and *M abscessus* group in 2 patients.

^c^None received treatment.

^d^Some patients had >1 rationale for modification.

^e^Two patients received both imipenem-cilastatin (with ceftaroline or ceftazidime) and meropenem (with ceftaroline) at various points in their treatment course.

Eight of the 19 patients (42%) in the treated *M abscessus* group were started on dual β-lactam therapy, with an even split between meropenem and imipenem-cilastatin (both 4 of 8 [50%]) in those instances. Nine patients (18%) were started on dual β-lactam therapy for an average of 108 days each, with carbapenems being paired with ceftaroline the majority of the time (8 of 9 [89%]). Seven patients (78%) started on dual β-lactam therapy demonstrated improved symptoms, 3 (33%) improved or partially improved radiologic change, and 5 (56%) sustained culture conversion to negative at the time of evaluation. Regarding reasons for stopping the initial dual β-lactam therapy, 3 (33%) did so due to adverse events, 2 (22%) went to hospice or died, 1 (11%) had to adjust agents due to new susceptibility data, 1 (11%) refused further therapy, and 2 (22%) finished their planned dual β-lactam–containing induction or treatment course.

During the study period, more than a quarter of treated patients (13 of 49 [27%]) received dual β-lactam therapy at some point (12 of 13 [92%] had *M abscessus* group infections) for an average of 122 days. Of these 13 patients, 6 (46%) had to stop due to adverse events, 3 (23%) went to hospice or died, 1 (8%) had to switch therapy due to updated susceptibility data, 1 (8%) refused further treatment, and 2 (15%) finished their planned dual β-lactam–containing induction or treatment course. Most patients receiving treatment in the study (39 of 49 [80%]) needed an initial regimen modification at some point, with the most common reasons for changes including efficacy/optimization (16 of 49 [33%]) and tolerance issues (22 of 49 [45%]). Of the 49 patients who received therapy, 36 (73%) demonstrated improved symptoms, 18 (37%) improved or partially improved radiologic change, and 19 (39%) sustained culture conversion to negative at the time of evaluation (Table 5). While 13 of 49 treated patients (27%) died or were lost to follow-up during the study time frame, no known deaths were primarily attributable to NTM infection.

**Table 5.** Clinical Outcomes Among Patients Receiving Treatment

Outcome	Patients, No.Total (%)
Death	
All cause	13/49 (27)
Within 30 d	0/49 (0)
Within 60 d	1/49 (2)
Symptomatic change	
No change/unknown	5/49 (10)
Improvement	36/49 (73)
Treated patients receiving any intravenous therapy	18/24 (75)
Treated patients receiving only oral therapy	18/25 (72)
Persistent symptoms	7/49 (14)
Worsening symptoms	1/49 (2)
Radiologic change	
Unknown/too early to tell	19/49 (39)
Improved or partially improved	18/49 (37)
Treated patients receiving any intravenous therapy	9/24 (38)
Treated patients receiving only oral therapy	9/25 (36)
Unchanged	10/49 (20)
Worsened	2/49 (4)
Microbiologic change	
No change/unknown	27/49 (55)
Sustained culture conversion to negative	19/49 (39)
Treated patients receiving any intravenous therapy	15/24 (63)
Treated patients receiving only oral therapy	4/25 (16)
Culture conversion with late relapse	0/49 (0)
Continued culture positivity	3/49 (6)

## DISCUSSION

To our knowledge, this is one of the largest published studies to date assessing the epidemiology and treatment of NTM infections in a population of oncology patients, especially for the relatively short inclusion period (approximately 3 years) compared wight other similar reports [8, 15]. While the concept is not entirely new, there is limited literature on NTM infections in the oncology population overall (particularly at a single tertiary care cancer center). This is an especially important area of examination given the uniqueness of these patients, who are often receiving chemotherapy and NTM-directed therapy concurrently, which for several reasons can be much more difficult to manage than NTM infection alone. Historically, oncology patients can be very difficult to treat given their underlying and treatment-related comorbid conditions and immune deficiency (both from the chemotherapy they are receiving and from their disease itself). Toxic effects from chemotherapy and NTM-directed treatment (such as cytopenias, nausea/vomiting, nephrotoxicity, and hepatotoxicity) can often overlap or be additive, making it difficult to truly delineate the offending agent and develop a new plan going forward.

Drug interactions take on even more prominence in these patients, as many agents used for the treatment of NTM infections inhibit or induce key enzymes responsible for the metabolism of chemotherapy medications or their associated supportive agents. Coordination of care is also much more complex, as specialists from multiple disciplines and/or institutions must work together to help patients navigate complicated chemotherapy and NTM-directed treatment schedules, all the while communicating effectively to make sure their treatment plans and goals of care for both issues are in alignment. What makes this analysis truly intriguing, though, is the use of dual β-lactam therapy in several patients, particularly with meropenem and not necessarily imipenem-cilastatin. In this instance, dual β-lactam therapy is thought to have synergistic effects through the mechanism of target redundancy [16–19]. LD-transpeptidases play an important part in cross-linkage of the peptidoglycan layer of the mycobacterial cell wall [17]. Both β-lactams in the combination target several LD-transpeptidases with additive effects, likely caused either by changing the makeup of the peptidoglycan layer via the amount and order of cell wall synthesis enzymes activated or by causing variations in the enzyme targets that better facilitate binding of the other β-lactam [17, 18].

This process results in the interruption of peptidoglycan synthesis in the NTM organism and subsequent reduction in its minimum inhibitory concentration [18]. The initial literature frequently demonstrated proof of this concept in clinical scenarios with imipenem-cilastatin [20–22]. In recent years, there has been exploration with meropenem in a similar role. Al-Jabri and colleagues [17] found that in vitro *M avium* complex susceptibility to meropenem was significantly enhanced with the addition of cefuroxime, ceftaroline, and cefdinir, with the effects being most pronounced with the latter 2 cephalosporins. From a clinical perspective, Becken and colleagues [19] described a young child with *M abscessus* group infection whose initial regimen included a meropenem-containing dual β-lactam combination (before therapy was later switched to another dual β-lactam tandem based on additional antimicrobial susceptibility testing), achieving both early clinical and microbiologic cure.

In our analysis, more than one-quarter of patients on treatment received dual β-lactam therapy at some point during their course of therapy, with many of them receiving a meropenem-based combination. There were no formal criteria for initiating dual β-lactam therapy in our analysis. While use was overwhelmingly driven by the susceptibility results of the offending organism, other factors, such as infection severity and extent, adverse events with prior therapies, drug interactions, and host factors (for example, cancer/comorbid conditions or concurrent chemotherapy) were also considered. We most commonly observed cytopenias, nausea/vomiting, hepatotoxicity, and nephrotoxicity in these patients (though it is worth mentioning that we could not always definitively conclude that dual β-lactam therapy was responsible, given the frequent presence of other potential contributors, such as chemotherapy, medications, comorbid conditions, and more). It has been demonstrated that imipenem-cilastatin may exhibit better coverage against *M abscessus* group than meropenem alone [23, 24]. The addition of dual β-lactam therapy (often ceftaroline in our analysis) can help overcome the deficit, allowing us to take advantage of some of the other benefits of a meropenem-based regimen—primarily its improved stability over imipenem-cilastatin and incorporation into outpatient parenteral antimicrobial therapy, where treatment can often last several months, or even longer in some cases [17, 25–30].

Given that our analysis was a single-center, descriptive, and noncomparative study, note that we cannot conclude with certainty that dual β-lactam therapy is effective or superior to other antimicrobial regimens until more trials are conducted in the future. In addition, further research is necessary to delineate several aspects of dual β-lactam therapy itself, including the optimal timing and duration of treatment, the role of testing for antimicrobial synergy with β-lactam combinations, preferred β-lactam agents, and the choice of additional antimicrobials to be used in the regimen, among others. That being said, the use of meropenem as part of dual β-lactam therapy is relatively new in the literature, and given its use and success in several of our cases it will continue to be explored at our institution as a potentially viable option for treating NTM infection.

Another pertinent aspect of our study to highlight involves the use of bedaquiline, a diarylquinolone sometimes used in the treatment of multidrug-resistant tuberculosis [31]. Building on this, several analyses have examined bedaquiline as an emerging option for treating NTM infections [31, 32]. To our knowledge this is one of the first examples in the literature of bedaquiline’s administration in our particular patient population. One patient in our study successfully received bedaquiline (400 mg by mouth every 24 hours for 2 weeks, then 200 mg by mouth every Monday, Wednesday, and Friday thereafter) upfront along with clofazimine and tigecycline for a 188-day course to treat a multidrug-resistant *M abscessus* group subspecies *abscessus* skin and soft-tissue infection (this patient had previous breast ductal carcinoma). Bedaquiline levels were obtained during therapy and found to be consistent with adequate concentrations in the plasma. Overall the patient tolerated the agent well and demonstrated symptomatic improvement of the affected area, along with sustained culture conversion to negative.

Another patient had bedaquiline introduced later in the course of therapy for a *M abscessus* group pulmonary infection after issues with tolerating several prior antimicrobials. Bedaquiline levels again reached adequate concentrations in the plasma during roughly 6 months of therapy with the agent. Further examination of bedaquiline is needed to make any definitive conclusions on its specific place in the NTM treatment hierarchy. However, given several issues associated with many typical NTM treatment medications (including antimicrobial resistance, adverse effects, and suboptimal outcomes) bedaquiline seems to be an intriguing option in certain clinical scenarios. While these were the only 2 patients receiving bedaquline in our study's time frame, our senior author has subsequently treated several more patients with the agent and continued to experience promising outcomes (A. P. C., personal communication, 25 March 2026). We hope to share the results of these cases in the future once more data are accumulated.

Treatment regimens in our study were heterogenous (in terms of the antimicrobials they contained and how long they were administered) depending on the organism involved, site of infection, tolerability, and several other factors. On the whole, however, most were fairly consistent with typical approaches in everyday practice and current guideline recommendations [10–13]. One exception (due to individual provider prescribing preferences) was a long duration of intravenous therapy. The 24 patients receiving agents parenterally received them for a median 90 days, with many receiving slightly longer intravenous courses than the typical duration in clinical practice in certain scenarios [33]. Notably, treated patients who received any therapy intravenously had numerically higher rates of improved, symptoms, improved or partially improved radiologic change, and sustained culture conversion to negative at the time of evaluation, compared with patients receiving only oral therapy. While this extended exposure to intravenous antimicrobials could explain some of the positive outcomes seen with the particular cases in our study, further research is needed to explore this concept and determine the ideal length of treatment with such agents in NTM.

Not all patients in our study received treatment. Clinicians were extremely judicious in deciding which patients were started on therapy, based on several factors, including symptoms, organism pathogenicity, the presence of concurrent oncologic disease (along with its severity, impact on the immune system, and prognosis), concomitant cancer treatment (with its related drug interactions and with its adverse effects, including ongoing cytopenias, immune suppression, hepatotoxicity, nausea/vomiting, and more), comorbid conditions, and patient goals of care related to quality of life and life expectancy [10, 11]. It is an important takeaway that sometimes, even in patients with an active NTM infection, watchful waiting may be the best approach when considering the entirety of these.

Limitations of this study include its retrospective design (hence the inability to control for all variables that could potentially influence the study's objectives) as well as selection bias. While its data set is among the largest to date on this particular patient population, our study still had a relatively small sample size, not large enough to make a definitive statement on the topic. With regard to epidemiology, given differences in presentation from a typical *M avium* complex infection, 2 cases of *M intracellulare* were classified as “other.” We acknowledge the limitation that each species so classified may be different. Separating each individual species in these few select cases would be extremely difficult (given that some infections were polymicrobial) and would also detract from the study's major focus on the much more prevalent NTM organisms.

Given that no current treatment guideline or protocol exists at our organization and the aforementioned paucity of data of NTM infections in oncologic patients, selection and evaluation of the effectiveness of antimicrobial therapy relied on the clinician's individual judgment. Poor clinical documentation in notes, collector error or misinterpretation, and lack of adequate patient follow-up could also make data collection more difficult, inaccurate, or incomplete. One patient in the study did have 2 distinct NTM infections treated with separate antimicrobial courses in different time periods (*M abscessus* group and subsequent macrolide-resistant *M avium* complex), which were counted as 2 cases to simplify data reporting.

In conclusion, our report offers one of the largest single-center epidemiologic perspectives regarding NTM infections in oncology patients. Cases at our institution were primarily caused by *M avium* complex or *M abscessus* group and were typically pulmonary, isolated by means of bronchoalveolar lavage. Looking at types of cancer, *M avium* complex was the most common pathogen in lymphoma and integument, lung, head and neck, and bladder cancer, while *M abscessus* group was the most common isolate in cases of leukemia, breast cancer, and multiple myeloma, among others. Our study expands on our initial abstract report and also offers unique insights into treatment practices in this population [34]. More than half of our patients received treatment, with most receiving a combination of ≥3 agents upfront. The majority of those treated required a modification to their regimen at some point during the course of therapy, most often due to tolerance or efficacy/optimization issues. Notably, several patients received dual β-lactam therapy with meropenem (in conjunction with ceftaroline) during the study period, a treatment approach that has been explored only relatively recently and demands further investigation in this population going forward.
